# Health risks encountered by Dutch medical students during an elective in the tropics and the quality and comprehensiveness of pre-and post-travel care

**DOI:** 10.1186/1472-6920-10-89

**Published:** 2010-12-02

**Authors:** Elhadi Sharafeldin, Darius Soonawala, Jan P Vandenbroucke, Evelien Hack, Leo G Visser

**Affiliations:** 1 Department of Infectious Diseases, Leiden University Medical Center, Albinusdreef 2, 2300 RC Leiden, Netherlands; 2 Department of Clinical Epidemiology, Leiden University Medical Center, Albinusdreef 2, 2300 RC Leiden, Netherlands; 3 Department of Student Affairs, Leiden University Medical Center, Hippocratespad 21, 2300 RC Leiden, Netherlands

## Abstract

**Background:**

Clinical and research electives abroad offer medical students many unique experiences. However, participating in an unfamiliar health-care setting combined with limited medical experience may place students at risk of illness. To improve pre-and post-travel care, we assessed the health risks and the quality and comprehensiveness of pre-and post-travel care in a cohort of Dutch medical students returning form an elective abroad.

**Methods:**

All medical students who had performed an elective in the tropics between July 2006 and December 2008 were sent an informative email asking them to complete a web-based questionnaire.

**Results:**

180 of 242 (74%) students completed the questionnaire. Regarding the risk of bloodborne viral infection: 67% of all students and 32% of junior students engaged in procedures that constitute a risk of exposure to bloodborne viral infection, often in countries with high HIV prevalence rates. None of nine students who experienced possible or certain mucosal or percutaneous exposure to potentially infectious body fluids reported the exposure at the time it occurred and none used PEP. Regarding other health risks: 8 of 40 (20%) students stopped using mefloquine due to adverse effects. This left a sizeable proportion unprotected in countries that are hyperendemic for malaria. Post-travel screening for schistosomiasis, tuberculosis (tuberculin skin test) and carriage of methicillin-resistant Staphylococcus aureus (MRSA) encompassed approximately half of all students who should have been screened.

**Conclusions:**

Based on the results of this study we have adopted an integral set of measures to reduce the health risks associated with an elective abroad. The pre and post-travel consult has been centralized and standardized as well as the distribution of PEP. In addition we have developed a mandatory module on Global Health for all medical students planning an elective abroad.

## Background

Clinical and research electives abroad offer medical students many unique experiences. Shouldering responsibility in a different health care system and working with underserved patients broadens the personal and medical horizon. This may even influence future career choice as international medical experience is associated with an increase in the choice for a primary care specialty [1]. A number of studies have surveyed the health risks facing students during an elective abroad and the pre-travel advice [2-9]. Particular regard has been given to the risk of bloodborne viral infection. For example, it is worrying that 75% of students fail to report exposures to potentially infectious body fluids [4].

Each year approximately 300 students enroll in the medical program at Leiden University Medical Center (LUMC) in The Netherlands. Approximately half of them perform one or more electives abroad. Unlike other medical schools, ours allows students to go on electives in countries where infection with Human Immunodeficiency Virus (HIV) is endemic and does not restrict senior students who have completed the fourth college year from performing surgical or obstetric practice in such countries. To receive study credits it is mandatory that the students obtain permission from the student registrar before departure. If study credits are obtained, it is also mandatory for students to seek a Dutch supervisor who assesses the quality of the planned elective and who judges the students' written report at the end of the elective. The registrar's office provides general information on preparation for an elective abroad and advises students to obtain pre-travel counseling and immunization. Although the university occupational health department provides such counseling and immunizations, the students are free to visit any other travel clinic including the LUMC in-hospital travel clinic or their general practitioner. As part of the travel advice, and depending on the destination and intended elective, the health department or travel clinic may refer the student to an infectious disease consultant for counseling on the need of carrying post-exposure prophylaxis for HIV (PEP) with them and on its use. Upon return home, no standard post-travel counseling is offered.

To improve pre-and post-travel care, we performed a questionnaire study of students returning from an elective abroad. We assessed the health risks and the quality and comprehensiveness of pre-and post-travel care. This led to improvements that are described in the discussion.

## Methods

All medical students who had performed an elective abroad between July 2006 and December 2008, who had visited countries where hepatitis A is endemic, and who had notified the student registrar to obtain study credits, were sent an informative email asking them to complete a web-based questionnaire. This study was designed in 2008. Students who had returned home prior to December 2007 were sent an email in May 2008. Students who returned between December 2007 and November 2008, which is during the conduct of this study, were sent an email in November 2008. Non-responders were sent a reminder two weeks after the first email. The questionnaire was designed to seek information on pre-travel preparation including vaccinations, on characteristics of the elective, on health risks (in particular the exposure to and protection against bloodborne viruses), on adherence to advice regarding anti-malarial measures and on illness while abroad and upon returning home. In addition the rate of routine screening for tuberculosis using one pre-and one post travel Tuberculin Skin Test (TST) was surveyed. We also surveyed the rate and result of screening for methicillin-resistant Staphylococcus aureus (MRSA) as students visiting foreign hospitals may import MRSA to Dutch hospitals. Finally we surveyed the rate and result of screening for schistosomiasis. The questionnaire was piloted among acquaintances and among staff of the department of Clinical Epidemiology at the Leiden University Medical Center. The protocol of this study (protocol 08/37B) was studied by the Medical Ethics Committee of Leiden University Medical Center in The Netherlands. The Medical Ethics Committee did not object to the conduct of this study.

## Results

The mean number of days between having completed the elective and completing the questionnaire was 235 days (interquartile range 121 to 325 days, range 2 to 638 days). The characteristics of the responders and of the electives are described in Table 1; 242 students were sent a questionnaire. Of the 180 (74%) who completed it the majority (78%) was female; 77% had planned a holiday before or after the elective, and the mean duration of the time spent abroad was 74 days (median 69 days, range 10 to 224 days). The majority went for the purpose of a clinical (47%) or pre-clinical elective (16%) as opposed to research or volunteer work (37%). Surinam was visited by 31%, making it the most popular destination. Obstetrics and gynecology (42%) was the most popular rotation. Before departure 90% consulted a center specialized in travel medicine; 4% sought advice from their general practitioner and 6% did not obtain advice from a qualified source.

**Table 1:** Baseline characteristics of 180 Dutch medical students returning from an elective abroad.

Parameter		
Mean age *years *(range)	23	(19-38)
Female (%)	141	(78)
College year *n *(%)		
Second	18	(10)
Third	39	(22)
Fourth	25	(14)
Fifth	27	(15)
Sixth	71	(39)
Type of elective *n *(%)		
Pre-clinical elective	29	(16)
Clinical elective	85	(47)
Research elective	60	(33)
Volunteer work	6	(3)
Mean duration of stay *days *(range)	74	(10-224)
Travel destination *n *(%)		
Africa	75	(42)
sub-Saharan Africa	54	
Malawi	16	
Cameroon	9	
Ghana	5	
Kenia	5	
Tanzania	4	
Uganda	4	
Other	11	
South-Africa	21	
Latin America	67	(37)
Surinam	56	
Other	11	
Asia	32	(18)
Nepal	11	
Indonesia	10	
China	6	
Other	5	
Middle-East	5	(3)
Eastern Europe	1	(1)
Holiday at the end of the elective *n *(%)	139	(77)

### Risk of infection with bloodborne viruses

All 180 students had been vaccinated against hepatitis B. The vaccine response is checked by the university occupational health department. For privacy reasons we did not have access to the response data; 120 students (67%) performed at least one type of procedure that is associated with an increased risk of exposure to bloodborne viral infection (i.e. surgical or obstetric practice, suturing, phlebotomy) (Table 2). In general, before completing the fourth college year, students have not yet been trained to perform many of these procedures. Therefore it is surprising that of the 58 junior students, 18 (32%) did take part in such activities. Procedures associated with an increased risk of exposure to bloodborne viral infection were also performed in countries with high HIV prevalence rates (Table 3). Some students received medical care while on elective which increases the risk of exposure to bloodborne viruses. Two students received dental care and ten received an intramuscular or intravenous injection.

**Table 2:** Number and percentage of 180 Dutch medical students who performed procedures associated with an increased risk of exposure to bloodborne viral infection during an elective abroad.

College year completed	2^nd ^(*n *= 18)	3^rd ^(*n *= 39)	4^th ^(*n *= 25)	5^th ^(*n *= 27)	6^th ^(*n *= 71)	All (*n *= 180)
Activity *n *(%)						
Obstetric practice	2 (11)	6 (15)	6 (24)	6 (22)	50 (70)	70 (39)
Surgical practice	1 (6)	13 (33)	10 (40)	16 (59)	58 (82)	98 (54)
Suturing	0	5 (13)	2 (8)	8 (30)	50 (70)	65 (36)
Phlebotomy	1 (6)	1 (3)	6 (24)	8 (30)	38 (53)	54 (30)
*Any of the above*	*3 (17)*	*15 (39)*	*15 (60)*	*19 (70)*	*68 (96)*	*120 (67)*
*None of the above*	*15 (83)*	*24 (62)*	*10 (40)*	*8 (30)*	*3 (4)*	*60 (33)*

Results stratified by college year.

**Table 3:** Number and percentage of 180 Dutch medical students who performed procedures associated with an increased risk of exposure to bloodborne viral infection during an elective abroad.

HIV prevalence rate	1-5% (*n *= 77)	5-15% (*n *= 23)	> 15% (*n *= 25)
Activity *n *(%)			
Obstetric practice	48 (62)	11 (48)	3 (12)
Surgical practice	57 (74)	9 (39)	8 (32)
Suturing	42 (55)	5 (22)	9 (36)
Phlebotomy	33 (43)	5 (22)	11 (44)
*Any of the above*	*66 (86)*	*14 (61)*	*15 (60)*
*None of the above*	*11 (14)*	*9 (39)*	*10 (40)*

Results stratified by adult HIV prevalence rates in the country where the elective was carried out.

Depending on type of elective, the destination and the on-site availability of antiretroviral drugs students were advised to take post-exposure prophylaxis with them; 31 students (17%) carried their own supply of PEP but 12 of these students need not have done so as they did not perform procedures that put them at risk of exposure to HIV. Of the 120 students who did perform such procedures, 66 (55%) either had onsite access to PEP or carried a personal supply; 51 (43%) did not know whether the hospital where they performed their elective had PEP and three students (2%) knew that they did not have onsite access to PEP.

Four students experienced mucosal or percutaneous exposure to potentially infectious body fluids while on elective (two in Surinam, one in South Africa and one in Malawi). Five students were unsure whether the event they had experienced qualified as such. None of the students had reported the exposure at the time it occurred and none had used PEP even though all except one either had onsite access to PEP or carried a personal supply. As a result of their response to the questionnaire these nine students were offered screening for HIV and hepatitis C. For reasons of confidentiality we could not find out whether these students opted to be screened.

### Other health risks

Nearly all students (98%) filled out the optional questions regarding sexual contact during the time abroad. Eight female students (6%) and three male students (8%) reported having had sex with a new partner; in seven instances with a partner native to the country where the elective was performed. We did not ask whether a condom was used.

Schistosomiasis may be acquired through fresh water contact; 76 students had swum or waded in fresh water in countries where schistosomiasis is prevalent. Of these students 22 had swum in highly endemic countries in sub-Saharan Africa. Eleven of these 22 students had consulted a physician upon return and had mentioned the fresh water contact, 10 were screened of which two showed seroconversion for antischistosomal antibodies.

One student reported a bite by an unidentified animal in the forest in Surinam. He was not vaccinated for rabies. Overall 28 students had been vaccinated against rabies prior to departure.

### Malaria chemoprophylaxis

The majority of students (83%) who visited areas that are endemic for malaria used a bed net. Of the 129 students who visited such areas nearly all were prescribed an adequate chemoprophylaxis (75 atovaquone-proguanil, 43 mefloquine, two proguanil, one primaquine and one doxycycline). One student had been prescribed chloroquine by a relative and six students did not remember which prophylaxis had been prescribed. Many students visited countries where malaria prophylaxis is only indicated for selective parts of the country. Of this group 17 did not start prophylaxis. In total 112 students started malaria chemoprophylaxis.

Of the 40 students who used mefloquine 18 (33%) reported an adverse effect: mainly sleep or mood disorder. One student returned prematurely due to neuropsychological adverse effects. Of the 62 students on atovaquone-proguanil 12 (19%) experienced an adverse effect: mainly gastro-intestinal complaints. Eight students who used mefloquine (20%) stopped the drug prematurely as did ten students on atovaquone-proguanil (16%) and the student on doxycycline. Only two of these students switched to another prophylaxis. One did so after having had malaria. All students who stopped using mefloquine did so due to adverse effects. Shortage of tablets or simply forgetting to take the prophylaxis constituted the main reasons for stopping the use of atovaquone/proguanil. Premature stopping of prophylaxis left eight students (15%) unprotected during part of their elective in hyperendemic regions in sub-Saharan Africa.

One student in Benin and one in Kenia were diagnosed with malaria. Both had used mefloquine, but the latter was one of those who had stopped the use due to side effects.

### Health problems

Diarrhea was the most common illness and was reported by 117 of 180 students (65%). The incidence was even higher (93%) among 40 students who did not have running water at their lodgings. Most cases were self-limiting and did not last beyond a week. However, 25 of 117 students (21%) had diarrhea accompanied by either bloody stools or fever, and in 29 of 117 students (25%) diarrheal illness caused a temporary interruption of the elective for a mean duration of 2.5 days (median 2 days, range 1 to 7 days). Thirteen of 117 students (11%) consulted a physician for diarrheal illness, three were admitted to hospital, and five received intramuscular or intravenous treatment.

Other common health problems were: constipation (33%), skin infections and wounds (29%) and upper respiratory tract infection (11%). Two students were involved in a traffic accident.

Twenty eight students used an antimicrobial agent; thirteen for enteritis, seven for a urinary tract infection and four each to treat a skin infection and respiratory tract infection.

### Post-travel

Seven students (4%) reported having had a fever shortly after returning home. Two of these students consulted a physician and one was diagnosed with Dengue. Travel-related illness after having returned home caused five of 180 students to interrupt their medical course for a period of 7 to 28 days; one due to Dengue, one due to neuropsychological problems attributed to the use of mefloquine, one due to an upper respiratory tract infection and two because they were identified as carriers of MRSA. Dutch hospitals have a low MRSA infection rate and adopt a strict policy to prevent spread of this bacterium [10]. Screening for MRSA using pharyngeal and nasal swabs is mandatory for hospital employees with recent employment abroad. Upon return, 79 of 180 students (44%) were screened of which two were found to be MRSA carriers (3%). The main focus of screening should be aimed at senior year students involved in clinical work; 70 of 121 senior year students (58%) had been screened for MRSA. Screening was mainly done at the instigation of hospital occupational health departments.

Depending on the destination and the duration of the elective, students are advised to have themselves tested for tuberculosis before departure and 8 weeks after returning home; 84 of 173 students (49%) had a TST performed after returning home. Two students (2%) had a positive reaction which had been negative before the elective abroad. Both had been on a clinical elective; one in Benin and one in Nepal. Both were referred to the municipal health service for counseling.

## Discussion

We assessed the health risks that face medical students on an elective abroad to improve the quality and comprehensiveness of pre-and post-travel care. A number of results are related to the risk of bloodborne viral infection. Firstly, we found that regardless of the study year the students were in, none took action following mucosal or percutaneous exposure to potentially infectious body fluids. This result is similar to that of a survey among British medical students [4]. Secondly, junior students on pre-clinical electives often took part in procedures that pose a risk for bloodborne viral infection. We were not informed about the individual capabilities of the students. Junior students may have had extra-curricular training to perform certain procedures before starting the elective, or they may have been supervised adequately during the elective while learning new procedures. Nevertheless, junior students have not yet received the standard curricular training and in general have limited clinical experience. This puts them at a greater risk of mucosal or percutaneous exposure to potentially infectious body fluids while performing procedures. They may also be less well informed how to act in case of such exposure. Thirdly, we found that allocation of PEP starter kits was inadequate. Kits were commonly handed out to students who turned out not to be at risk of coming in contact with potentially infected body fluids and were not handed out to a sizeable group of students who may have been at risk of such exposure. Due to the difficulty in predicting what students will do while on the elective, improving the pre-travel assessment of who should carry a PEP starter kit is not straightforward. Lastly, systematic education on safe sex should be stressed, as 6% of the students reported that they had sex with a new partner while abroad.

This survey also detected other health risks. One in five students stopped using mefloquine due to adverse effects, which means that a sizeable proportion was left unprotected against malaria. Diarrheal illness was very common as is to be expected. Importantly, a small proportion needed to be hospitalized or required intramuscular or intravenous treatment for diarrheal illness. We also found that medical care following return from the elective can be improved upon. Screening for schistosomiasis, tuberculosis and MRSA did not encompass all who should have been screened.

This study has a number of strengths and limitations. It was restricted to students who had applied for study credits, and we expect this group to constitute the majority of students who perform an elective abroad. For a web-based questionnaire, the response rate was relatively high and none of the questionnaires was incomplete. There are two limitations. The survey was not completely anonymous as we asked the age, gender, study year and e-mail address of the participants. This may have prompted socially desirable answers. The time between having completed the elective and filling out the questionnaire was not standardized and was sometimes quite long which may have reinforced recall bias. To reduce the chance of such bias, we mainly surveyed events that are unlikely to be forgotten, such as needle-stick injury, malaria and diarrheal illness.

### Measures that are intended to limit the health risks associated with an elective abroad

Based on the results of this study a number of measures have been adopted to reduce the health risks associated with an elective abroad. Firstly, it has been made mandatory that all medical students planning an elective abroad follow a module on Global Health prior to departure [11-13]. The aim of this module is to enhance student safety and student learning, and to highlight the ethical dimension of an elective abroad. Secondly, at the visit to the administrative department all students are now strongly advised to visit the university occupational health department instead of opting to visit another travel clinic or the general practitioner. By centralizing pre-travel advice, as has been suggested by Tilzey and Banatvala [14] we expect to achieve a number of improvements. The risk of bloodborne viral infection and the on-site availability of PEP are systematically assessed. This assessment has been standardized. We now ask students to fill out a form describing which procedures they plan to perform. This form is signed by the Dutch supervisor, who judges whether the student is competent to perform the planned procedures and who judges whether the student will be adequately supervised during the elective in case he/she is to learn a new procedure. Based on this signed form, an assessment can be made during the pre-travel consult whether PEP needs to be provided. Whereas students first had to pay for their PEP kit, it is now provided at no cost by the university. To reduce the threshold for reporting and acting on an exposure to potentially infectious body fluids, the written information has been adapted. It now contains a checklist that specifies which steps to take in case of exposure.

If a traveler is to experience adverse effects when using mefloquine, such effects often manifest in the first few weeks of usage. Therefore it is common policy to prescribe mefloquine on trial prior to departure. By centralizing pre-travel advice we aim to increase the proportion of students that receive mefloquine on trial. We have also adapted the written information. In case of adverse effects which seem attributable to mefloquine, students are advised to use half the dosage twice weekly instead of the standard full dosage once a week in order to lower the peak plasma concentration [15]. Furthermore, students are urged to contact the on call infectious disease consultant in our hospital if they are considering stopping chemoprophylaxis.

To improve post-travel care, upon return all students must now fill out a standard short web-based checklist which assesses certain health risks (exposure to potentially infected body fluids, the risk of schistosomiasis and the need for screening for tuberculosis and MRSA). This results in a computer generated recommendation which states whether the student needs to contact the occupational health department or another care provider for a post-travel consult.

## Conclusion

Many of the health risks that were detected in this survey are probably not unique to Dutch medical students. We believe that adopting a standardized pre-and post-travel consult will reduce these health risks by reinforcing knowledge regarding prevention of bloodborne viral infection, by maintaining a clear-cut policy on provision of PEP, by addressing the problem of treatment limiting adverse events with regard to malaria prophylaxis, by reducing the chance of (latent) tuberculosis and chronic schistosomiasis and by preventing spread of MRSA. In a future survey we intend to see whether the new policy is indeed effective in protecting our medical students by limiting health risks.

## List of abbreviations

(LUMC): Leiden university Medical Center; (HIV): Human Immunodeficiency Virus; (PEP): post-exposure prophylaxis for HIV; (TST): Tuberculin Skin Test; (MRSA): methicillin-resistant Staphylococcus aureus

## Competing interests

The authors declare that they have no competing interests.

## Authors' contributions

ES participated in the preparation of the protocol and in data acquisition and in revising the manuscript. DS participated in the data analysis and interpretation of the data and in writing the manuscript. JPV participated in the preparation of the protocol and in interpretation of the data and in revising the manuscript. EH participated in the preparation of the protocol and in data acquisition and in revising the manuscript. LGV conceived the study, participated in the preparation of the protocol, interpretation of the data and in revising the manuscript. All authors read and approved the final manuscript."

## Funding

There was no dedicated funding for this project.

## Pre-publication history

The pre-publication history for this paper can be accessed here:

http://www.biomedcentral.com/1472-6920/10/89/prepub

## References

[B1] Grudzen CR Legome E Loss of international medical experiences: knowledge, attitudes and skills at risk BMC Med Educ 2007 7 47 10.1186/1472-6920-7-47 18045481 PMC2242732

[B2] Philipp R Webber S Butler AV Macara AW Student health during overseas electives J R Coll Gen Pract 1985 35 80 83 3989772 PMC1959941

[B3] Inglis TJJ Travel-Associated Health Risks of Singaporean Medical Students J Travel Med 1996 3 80 82 10.1111/j.1708-8305.1996.tb00710.x 9815429

[B4] Gamester CF Tilzey AJ Banatvala JE Medical students' risk of infection with bloodborne viruses at home and abroad: questionnaire survey BMJ 1999 318 158 160 9888908 10.1136/bmj.318.7177.158 PMC27693

[B5] Moss PJ Beeching NJ Provision of health advice for UK medical students planning to travel overseas for their elective study period: questionnaire survey BMJ 1999 318 161 162 9888909 10.1136/bmj.318.7177.161 PMC27694

[B6] Abdullah ASAMM Hedley AJ Fielding R Prevalence of travel related illness amongst a group of Chinese undergraduate students in Hong Kong J Travel Med 2000 7 125 132 10.2310/7060.2000.00043 11179941

[B7] Cossar JH Allardice GM Whiting B Health surveillance of Glasgow medical undergraduates pursuing elective studies abroad (1992-1998) J Travel Med 2000 7 314 318 10.2310/7060.2000.00085 11305241

[B8] Franklin GF Gray K Nathwani D Provision of drugs for post-exposure prophylaxis of HIV for medical students on overseas electives J Infect 2001 43 191 194 10.1053/jinf.2001.0903 11798258

[B9] Goldsmid JM Bettiol SS Sharples N A preliminary study on travel health issues of medical students undertaking electives J Travel Med 2003 10 160 163 10.2310/7060.2003.35728 12757690

[B10] Vandenbroucke-Grauls CM Methicillin-resistant Staphylococcus aureus control in hospitals: the Dutch experience Infect Control Hosp Epidemiol 1996 17 512 513 10.1086/647355 8875295

[B11] Banatvala N Doyal L Knowing when to say "no" on the student elective. Students going on electives abroad need clinical guidelines BMJ 1998 316 1404 1405 9572746 10.1136/bmj.316.7142.1404 PMC1113113

[B12] Miranda JJ Yudkin JS Willott C International Health Electives: Four years of experience Travel Med Infect Dis 2005 3 133 141 10.1016/j.tmaid.2004.09.003 17292031

[B13] Edwards R Piachaud J Rowson M Miranda J Understanding global health issues: are international medical electives the answer? Med Educ 2004 38 688 690 10.1111/j.1365-2929.2004.01849.x 15200392

[B14] Tilzey AJ Banatvala JE Protection from HIV on electives: questionnaire survey of UK medical schools BMJ 2002 325 1010 1011 10.1136/bmj.325.7371.1010 12411360 PMC131018

[B15] van Riemsdijk MM Ditters JM Sturkenboom M Neuropsychiatric adverse events are associated with increased serum levels of mefloquine *PhD Thesis* Erasmus University Rotterdam, Department of Epidemiology and Biostatistics 2001

